# Early assessment by FDG-PET/CT of patients with advanced renal cell carcinoma treated with tyrosine kinase inhibitors is predictive of disease course

**DOI:** 10.1186/1471-2407-12-162

**Published:** 2012-05-02

**Authors:** Daiki Ueno, Masahiro Yao, Ukihide Tateishi, Ryogo Minamimoto, Kazuhide Makiyama, Narihiko Hayashi, Futoshi Sano, Takayuki Murakami, Takeshi Kishida, Takeshi Miura, Kazuki Kobayashi, Sumio Noguchi, Ichiro Ikeda, Yoshiharu Ohgo, Tomio Inoue, Yoshinobu Kubota, Noboru Nakaigawa

**Affiliations:** 1 Department of Urology, Yokohama City University Graduate School of Medicine, 3-9 Fukuura kanazawaku, Yokohama, 236-0004, Japan; 2 Department of Urology, Yokohama Sakae Kyosai Hospital, Yokohama, Japan; 3 Advanced Medical Research Center, Yokohama City University, Yokohama, Japan; 4 Department of Radiology, Yokohama City University Graduate School of Medicine, Yokohama, Japan; 5 Department of Urology, Kanagawa Cancer Center, Yokohama, Japan; 6 Department of Urology, Yokosuka Kyosai Hospital, Yokosuka, Japan; 7 Department of Urology, Yokohama Minami Kyosai Hospital, Yokosuka, Japan

## Abstract

**Background:**

We reported previously that ^18^F-2-fluoro-2-deoxyglucose positron emission tomography/ computed tomography (FDG PET/CT) had potential for evaluating early response to treatment by tyrosine kinase inhibitors (TKIs) in advanced renal cell carcinoma (RCC). This time we investigated the relation of the early assessment by FDG PET/CT to long-term prognosis with an expanded number of patients and period of observation.

**Methods:**

Patients for whom TKI treatment for advanced RCC was planned were enrolled. FDG PET/CT was performed before TKI treatment and after one month of TKI treatment. The relations of the FDGPET/CT assessment to progression free survival (PFS) and overall survival (OS) were investigated.

**Results:**

Thirty-five patients were enrolled (sunitinib 19 cases, sorafenib 16 cases). The patients with RCC showing high SUVmax in pretreatment FDG PET/CT demonstrated short PFS (*P* =0.024, hazard ratio 1.137, 95% CI 1.017-1.271) and short OS (*P* =0.004, hazard ratio 1.210 95% CI 1.062-1.379). Thirty patients (sunitinib 16 cases, sorafenib 14 cases) were evaluated again after 1 month. The PFS of the patients whose SUVmax decreased<20% was shorter than that of the patients whose SUVmax decreased<20% (*P* = 0.027, hazard ratio 3.043, 95% CI 1.134-8.167). The PFS of patients whose tumor diameter sum increased was shorter than that of the patient with tumors whose diameter sum did not (*P* =0.006, hazard ratio 4.555, 95% CI 1.543-13.448).

The patients were classified into three response groups: good responder (diameter sum did not increase, and SUVmax decreased ≥ 20%), intermediate responder (diameter sum did not increase, and SUVmax decreased<20%), and poor responder (diameter sum increased, or one or more new lesions appeared). The median PFS of good, intermediate, and poor responders were 458 ± 146 days, 131 ± 9 days, and 88 ± 26 days (good vs. intermediate *P* = 0.0366, intermediate vs. poor *P* = 0.0097, log-rank test). Additionally the mean OSs were 999 ± 70 days, 469 ± 34 days, and 374 ± 125 days, respectively (good vs. intermediate *P* = 0.0385, intermediate vs. poor *P* = 0.0305, log-rank test).

**Conclusions:**

The evaluation of RCC response to TKI by tumor size and FDG uptake using FDG PET/CT after 1 month can predict PFS and OS.

## Background

Renal cell carcinoma (RCC) accounts for 3% of all adult cancers [1]. Approximately 30% of patients are diagnosed with metastases and an additional 20-40% of patients develop metastases after radical nephrectomy with curative intent [2,3]. Cytokine therapies were the only systematic treatments available for advanced RCC for a long time, and the outcome of patients with metastatic RCC has been poor, with a median survival time of 10 to 21 months [4,5].

Recently, the oncogenic mechanism of RCC has been elucidated and drugs that target relevant biological pathways have been developed. Tyrosine kinase inhibitors (TKIs) such as sunitinib and sorafenib which target vascular endothelial growth factor (VEGF) receptors improved the prognosis of patients with metastatic RCC [6,7]. The antitumor activity of TKIs is not cytotoxic, like classical antitumor therapeutics, but rather cytostatic, suppressing biological activity by inhibiting angiogenesis. Practically, some RCCs treated with the TKIs do not decrease in tumor volume but enter a period of long-term dormancy, without enlargement of volume or novel metastasis. It has been suggested that a new assessment focusing not only on the volume of the tumors, but also biological activities to evaluate the antitumor activity of TKIs is necessary.

^18^F-2-fluoro-2-deoxyglucose positron emission tomography/ computed tomography (FDG PET/CT) is a useful non-invasive tool to evaluate glucose metabolic status, which can be the index of biological activity of cancer. Although PET has not been generally used for the screening of RCC due to the urinary excretion of the radiotracer, which can mask the presence of primary lesions [8,9], several investigators have reported recently that FDG-PET/CT had sufficient potential to evaluate advanced RCCs [10-12]. We previously reported the potential of FDG PET/CT as a tool to evaluate the early response to TKIs in advanced RCC, but the number of cases was small and the observation period was short [13]. This time, we investigated the potential with an expanded number of patients and period of follow-up.

## Methods

### Patients

Thirty-five patients were enrolled in this study. Patients had to be referred to Yokohama City University before the start of their treatment, from June 2008 until March 2011. This was a prospective study to clinically follow enrolled patients planning to undergo TKI therapy for advanced RCC. The pathologies of enrolled cases were confirmed by prior nephrectomy or biopsy. Patients with uncontrolled diabetes mellitus (blood glucose level >150 mg/dL) , with other known malignancies, and those treated with therapeutics during the 2 weeks prior to the scan were excluded. The study protocol was approved by the Yokohama City University Institutional Review Board. Written informed consent was obtained from all patients. The decision for patients to undergo therapy was made before the evaluation by PET/CT.

### Treatment

Sunitinib was given orally once a day at the dose of 50 mg in 6-week cycles consisting of 4 weeks of treatment followed by 2 weeks without treatment. Oral sorafenib 800 mg was given daily. The dose of sunitinib was reduced to 37.5 or 25 mg and that of sorafenib was reduced to 600 or 400 mg according to pretreatment general condition or major adverse events during treatment. Treatment was continued until disease progression, unacceptable adverse events, request by the patient, or surgery including nephrectomy.

### Imaging

Patients fasted for at least 6 hours prior to intravenous injection of ^18^F FDG. PET/CT images were obtained using a PET/CT system (Aquiduo 16; Toshiba Medical Systems, Tokyo, Japan). PET/CT images were acquired from the top of the head to the mid thigh at 60 min after intravenous injection of 2.5 MBq/kg of [^18^F] FDG. A low-dose non-contrasted CT scan was acquired first and used for attenuation correction. Emission images were acquired in 3-dimensional mode for 2 min per bed position. After PET acquisition, contrast-enhanced CT was performed with a 2-mm slice thickness, 120 kV, 400 mA, 0.5 s/tube rotation, from the top of the head to the mid thigh, with breath holding. A total of 100 ml contrast medium (iopamidol) was administered intravenously at a rate of 1.0 ml/s. The scan delay was set at 120 s after starting the injection of contrast material. The patients with serum creatinine level >1.5 mg/dL were examined without contrast material. Images were reconstructed by attenuation-weighted ordered-subset expectation maximization (OSEM) (four iterations, fourteen subsets, 128 ´ 128 matrix, with 5-mm Gaussian smoothing). The standardized uptake value (SUV) was determined according to the standard formula, with activity in the volume of interest (VOI) recorded as Bq per ml / injected dose in Bq per weight (kg). The maximum SUV (SUVmax) was recorded using the maximum pixel activity within the VOI. To obtain the SUVmax of the individual patient, the SUV of all lesions in tumors diagnosed as RCC by CT imaging were analyzed.

### Statistical analysis

Cox proportional hazards model was used to assess the impacts of pretreatment SUVmax, SUVmax change ratio, total diameter change ratio, and clinical parameters on progression-free survival. The progression-free survival and overall survival curves were estimated by the Kaplan-Meier method, and the resulting curves were compared using the log-rank test.

The statistical difference of SUVmax and SUVmax change between clear cell carcinoma and papillary carcinoma was determined by two-side Mann-Whiteney’s *U*-test. All statistical analyses were carried out with SPSS software (SPSS, Inc, Chicago, IL). Significance was assigned at P<0.05.

## Results

### Patients characteristics and intervention

Thirty-five patients were enrolled in this prospective study and evaluated by FDG PET/CT before treatment with TKIs (sunitinib 19 cases, sorafenib 16 cases). When the highest lesion SUV in individual patients was defined as SUVmax, SUVmax of the 35 patients ranged between 2.3 and 16.6 (mean 9.0). The patients with RCC tumors showing high SUVmax demonstrated short progression-free survival (PFS) (*P* = 0.024, hazard ratio 1.137, 95% CI 1.017-1.271) and short overall survival (OS) (*P* =0.004, hazard ratio 1.210 95% CI 1.062-1.379).

Thirty patients (sunitinib 16 cases, sorafenib 14 cases) were evaluated again after 1 month of treatment; the other, 5 patients (4 clear cell and 1 sarcomatoid) demonstrated deterioration of general status due to rapid progression within 1 month. The SUVmax range of the 5 patients was 8.9-16.6 (mean 14.1). The clinical characteristics of the 30 patients are detailed in Table 1. There were 25 men and 5 women. The mean age was 64 years (range, 32–80). Of all 30 patients, 23 had pure clear cell carcinoma, 5 had papillary carcinoma, 1 had clear cell carcinoma mixed with sarcomatoid component, and 1 long-term dialysis patient had a heterogeneous pathology with clear cell type and papillary type. The mean SUVmax was 8.1 (range, 2.3-16.1). The mean SUVmax of 23 pure clear cell carcinoma was 7.6(range, 2.3-14.8) and the mean SUVmax of 5 papillary carcinoma was 9.7 (range, 3.9-16.1). There was not statistical difference (*P* =0.413). The SUVmax of clear cell/sarcomatoid was 9.1. The SUVmax of the celar cell/papillary was 9.5. According to Memorial Sloan-Kettering Cancer Center (MSKCC) classification [14], one patient had favorable risk, 21 intermediate risk, and 8 poor risk. Twenty-two patients had undergone nephrectomy. Nineteen patients had no previous systematic therapies. Three patients had been treated previously with sorafenib and the treatment ended more than 1 month before the pretreatment evaluation by FDG PET/CT. Nine patients had previously been treated by IFN-alpha, and 2 by chemotherapy.

**Table 1:** Characteristic of 30 patients

**Age (year)**	**32-80 (mean 64)**
Gender	
Male	25
Female	5
Histology	
Clear cell	23
Papillary	5
Clear/Sarcomatoid	1
Clear/Papillary	1
TKI treatment	
Sunitinib	16
Sorafenib	14
MSKCC classification	
Favorable	1
Intermediate	21
Poor	8
Nephrectomy	
Yes	22
No	8
Prior treatment	
Non	19
IFN-α	9
Sorafenib	3
Chemotherapy	2

### Clinical outcome of 30 patients

The mean duration of observation was 458 days (range, 67–1114). At the date of analysis, 18 patients showed progressive disease (PD) as evaluated by RECIST version 1.1 and 10 patients had died due to progression of RCC. No patients had died for other reasons. The median PFS was 209 days (range, 27–887). Three patients (sunitinib, 2; sorafenib, 1) underwent nephrectomies after TKI treatment. Of the 14 patients treated with sorafenib, 10 patients changed to sunitinib due to PD, and 4 of the 10 patients changed to everolimus sequentially. One of the 14 sorafenib-treated patients changed directly to everolimus. Of the 16 patients treated with sunitinib, 4 patients changed to everolimus and 1 patient changed to sorafenib due to PD.

The impacts of some clinical parameters on PFS were analyzed by Cox proportional hazards modeling (Table 2). There was statistical difference only between the patients with liver metastasis and the patients without liver metastasis (*P* =0.004).

**Table 2:** Univariate Cox progression-free survival analyses of various clinical parameters

	**Univariate analysis**		
**Clinical Parameters**	**P-value**	**HR**	**95%CI**
sunitinib vs. sorafenib	0.341	1.585	0.614-4.096
clear cell vs. papillary	0.087	2.841	0.860-9.379
nephrectomy: yes vs. no	0.620	0.725	0.203-2.590
pretreatment: yes vs. no	0.205	0.500	0.171-1.459
previous TKI: yes vs. no	0.380	0.510	0.113-2.293
previous IFN: yes vs. no	0.056	0.284	0.078-1.033
number of lesions: 1–2 vs. 3 ≥	0.056	3.046	0.971-9.559
lung metastasis: only vs. others	0.359	0.552	0.155-1.967
bone metastasis: no vs. yes	0.927	0.942	0.264-3.365
liver metastasis: no vs. yes	0.004	7.672	1.891-31.130

### The assessment by FDG PET/CT

In pretreatment FDG PET/CT of the 30 patients who underwent two-time assessment, FDG accumulation was detected in 95 lesions of 107 lesions (89%) whose diameters were 1.0 cm or more. The mean number of RCC lesions in the individual patients was 3.5 (range, 1–9). The median date of the second FDG PET/CT after TKI treatment started was day 31 (range, 27–47). The median SUVmax in the second FDG PET/CT was 7.1 (range, 3.7-15.5). The mean ratio of SUVmax change compared with pretreatment FDG PET/CT was −18% (range, -55 to 65%). The mean ratio of the diameter change was −6% (range, -30 to 30%). No lesion remitted completely. A new lesion appeared in only 1 patient. The mean ratio of SUVmax change in clear cell carcinoma was −14.0%(range, -54.9%- 65.2%), and that in papillary carcinoma was −1.1%(range, -35.4%- 15.7%). The mean ratio of the diameter in in clear cell carcinoma was −5.7%(range, -30.2%- 29.7%), and that in papillary carcinoma was −6.5%(range, -22.4%- 13.8%). The ratios of SUVmax change and diameter change were not statistically different between clear cell carcinoma and papillary carcinoma (SUVmax change: p = 0.193, diameter change: p = 0.954).

According to the European Organization for Research and Treatment of Cancer (EORTC) criteria [15], in which the SUV cut-off point is 25%, 9 patients had a partial metabolic response, 14 patients had SD, and 7 had PD. None achieved complete remission (CR). There was no statistical association between the evaluation by EORTC criteria and PFS. However, the PFS of the patients whose tumor SUVmax decreased<20% after 1 month was shorter than that of those whose tumor SUVmax decreased<20% (*P* = 0.027, Cox hazard ratio 3.043, 95% CI 1.134-8.167). Additionally, the PFS of patients whose tumor diameter sum increased after 1 month was shorter than that of the patients whose tumors diameter sum did not increase (*P* = 0.006, hazard ratio 4.555, 95% CI 1.543-13.448).

Using these two predictive factors, we defined new and simple criteria for evaluating tumor response to TKI of advanced RCC as follows: good responder, diameter sum does not increase and SUVmax decreases<20%; intermediate responder, diameter sum does not increase and SUVmax decreases<20%; and poor responder, diameter sum increases or one or more new lesions appear.

**Figure 1:**
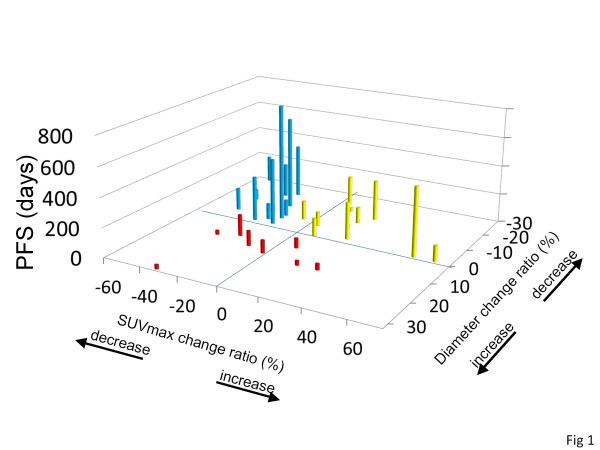
**The association between PFS and change ratios of SUVmax and the tumor diameter sum after 1 month in individual patients.** The information about PFS (days) was added as height to the 2-dimensional graph demonstrating the change ratios of SUVmax (horizontal axis) and those of tumor diameters sum (vertical axis) after 1 month in individual patients. Blue bars show the PFSs of good responders (diameter sum does not increase and SUVmax decreases ≥ 20%), yellow bars indicate those of intermediate responders (diameter sum does not increase and SUVmax decreases<20%), and red bars indicate those of poor responders (diameter sum increases or new lesions appear).

**Figure 2:**
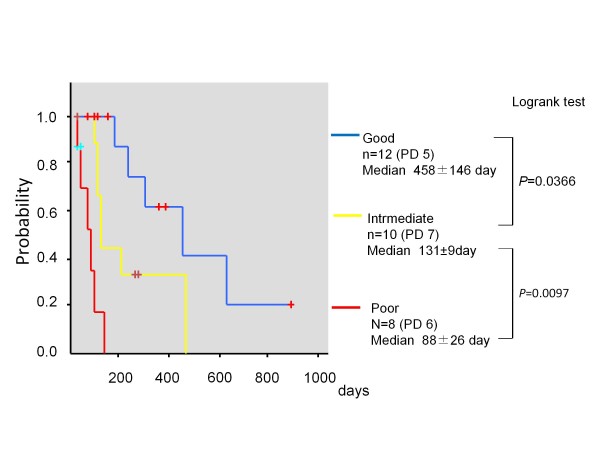
Kaplan-Meier curves of progression-free survival in 30 patients according to our response criteria.

**Figure 3:**
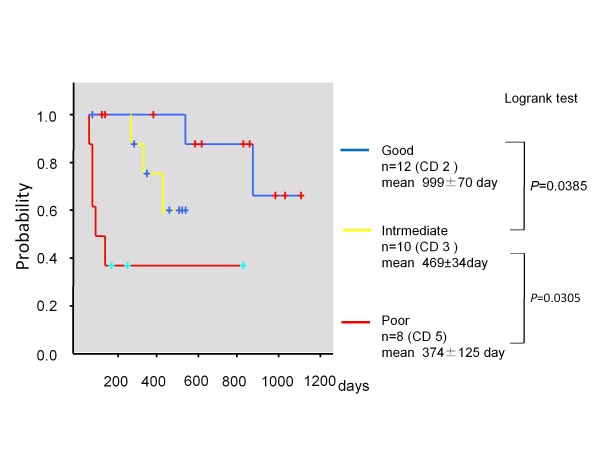
Kaplan-Meier curves of overall survival in 30 patients according to our response criteria.

According to the new criteria, the 30 patients were classified into 12 good responders (blue bars in Figure 1), 10 intermediate responders (yellow bars in Figure 1), and 8 poor responders (red bars in Figure 1). The median PFS of good, intermediate, and poor responders were 458 ± 146 days, 131 ± 9 days, and 88 ± 26 days, respectively. There was a statistical difference among these three groups (good vs. intermediate *P* = 0.0366, intermediate vs. poor *P* = 0.0097, log-rank test) as shown in Figure 2. Additionally, the mean OS of good, intermediate, and poor responders were 999 ± 70 days, 469 ± 34 days, and 374 ± 125 days, respectively (good vs. intermediate *P* = 0.0385, intermediate vs. poor *P* = 0.0305, log-rank test) as shown in Figure 3.

## Discussion

**Figure 4:**
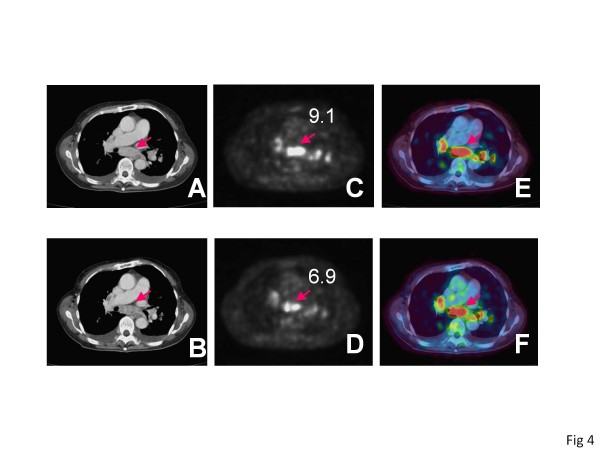
**A patient demonstrating long tumor dormancy.** 57 y.o. female with mediastinal lymph node metastasis. CT images at (A) pretreatment state and (B) post-treatment state, PET images at (C) pretreatment state and (D) post-treatment state, fused PET/CT images at (E) pretreatment state and (F) post-treatment state. The diameter of mediastinal lymph node decreased 7% and its SUVmax decreased 20% in post-sunitinib treatment state. She maintained an SD for 887 days.

This work demonstrated that the early assessment of response to TKIs using a combination of FDG uptake and tumor size could predict not only the PFS but also the OS of patients with advanced RCC. To our knowledge, it is the first to address this issue. The benefit of assessment by FDG uptake as well as is the ability to evaluate the biological dormancy induced by the treatments. Although the prognosis of advanced RCC was dramatically improved with the development of TKIs such as sunitinib or sorafenib, the best responses were usually found in stable diseases according to RECIST. Indeed, treatment by sunitinib is associated with 31% overall response rate and that by sorafenib was only 2% in clinical trials [6,7]. Some RCCs treated with TKIs do not decrease in tumor volume but maintain long-term dormancy without enlargement of volume or novel metastasis. But there have been no clinical answers to the question of whether an individual case treated with TKIs whose tumors did not decrease in size should continue the treatment or change to other therapeutic options including other TKIs and mammalian target of rapamycin (mTOR) inhibitors, which have been reported to benefit patients with advanced RCC [16,17]. This was primarily because there has been no biological marker to evaluate the biological activity of RCCs, especially the dormancy induced by TKIs. In this regard, FDG PET/CT is a useful tool to evaluate glucose metabolic status, which can be the index of biological activity of cancer; a decrease of FDG accumulation can express the biological dormancy of RCC induced by TKI treatment. Indeed, a patient with only 7% decrease in tumor diameter sum and 20% decrease in SUVmax maintained an SD status for 887 days (Figure 4) and another with only 3% decrease in the tumor diameter sum and 20% decrease in SUVmax achieved 458 days of SD in our series.

**Figure 5:**
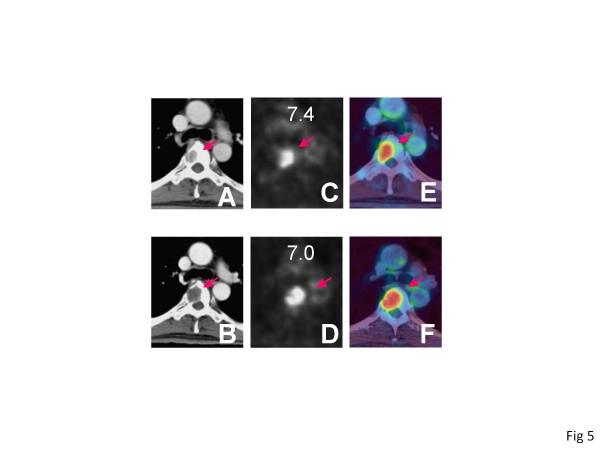
**A patient demonstrating rapid progression.** 59 y.o. male with thoracic vertebral metastasis. CT images at (A) pretreatment state and (B) post-treatment state, PET images at (C) pretreatment state and (D) post-treatment state, fused PET/CT images at (E) pretreatment state and (F) post-treatment state. The CT images showed that the metastatic lesion had homogeneous contrast enhancement at pretreatment status and the enhancement was attenuated apparently in post-sorafenib treatment. The SUVmax decreased only 5%. He died on day 88.

There have been trials to evaluate the tumor dormancy other than by tumor volume. Krajewski KM et al. previously reported that tumor attenuation in contrast-enhanced CT (CECT) predicts the outcome of RCC patients treated with sunitinib [18,19]. In our study, most patients whose tumors demonstrated attenuation had a decrease in SUVmax and their prognoses proved to be good. However, we experienced one case in which tumors showed apparent attenuation of contrast-enhancement but a decrease of only 5% in FDG uptake, and his prognosis was poor (Figure 5). It was speculated that some RCCs continue to progress against the inhibition of angiogenesis, and that attenuation was not a sufficient condition to reflect tumor dormancy. Additionally, the renal function of most patients with advanced RCC is deteriorated due to nephrectomy or the existence of the original tumor, and CETC entails a risk of worsening kidney function. So, we think that the evaluation by FDG PET/CT is more precise and safer than that by CECT.

However, it was not sufficient to assess the response to TKIs of RCCs by FDG uptake alone because there were some RCC cases with decreased SUVmax but increased tumor volume, and their prognoses were poor as shown Figure 1. In our series, 3 patients demonstrated a decrease in SUVmax ≥ 20% but an increase in tumor size after 1 month, and their PFSs were very short: 28, 28, and 146 days, respectively. Additionally, the classification of our series by EORTC criteria, which assess the response by FDG uptake, was not statistically associated with prognosis. Kayani et al. previously reported that the assessment of patients with RCC treated with sunitinib by FDG uptake alone after 4 weeks failed to predict the disease course [20]. These results suggested the existence of RCCs that progress independently of glucose uptake, and emphasize the need for a combination assessment of both tumor size and FDG uptake.

Some questions remain. We defined metabolic response as a ≥20% reduction of SUVmax in the novel criteria we advocated. The EORTC proposed a SUV diminution of 15-25% for a partial response after the first cycle. Kayani et al. the defined cut-off point as 20% when they evaluated the response to sunitinib in metastatic RCC and succeeded in predicting the disease course by assessment after 16 weeks. Lyrdal et al. reported that responders with a decrease of ≥20% FDG accumulation to sorafenib had a long OS. A further study expanding the number of patients is necessary to verify this cut-off point.

## Conclusion

The evaluation of RCC response to TKI treatment by tumor size and FDG uptake using FDG PET/CT after 1 month can predict not only the duration of response to TKIs, but also survival duration. Early assessment by FDG-PET/CT provides useful information to determine strategies for individual patients with advanced RCC. However, larger studies are needed to confirm these preliminary results.

## Competing interests

The authors declare that they have no competing interests.

## Authors’ contributions

NN had full access to all the data in the study and takes responsibility for the integrity of the data and the accuracy of the data analysis. All authors read and approved the final manuscript. Study concept and design: DU, MY, UT, RM, TI, YK, NN. Acquisition of data: KM, KN, TM, TK, KK, SN, II, YO. Analysis and interpretation of data: DU, MY, NN. Administrative, technical, or material support: TI, YK, NN. Drafting of the manuscript: DU. Critical revision of the manuscript for important intellectual content: NN. Obtaining funding and supervision: NN.

## Pre-publication history

The pre-publication history for this paper can be accessed here:

http://www.biomedcentral.com/1471-2407/12/162/prepub
